# Unconventional T cells in brain homeostasis, injury and neurodegeneration

**DOI:** 10.3389/fimmu.2023.1273459

**Published:** 2023-10-03

**Authors:** Mengfei Lv, Zhaolong Zhang, Yu Cui

**Affiliations:** ^1^ Institute of Neuroregeneration and Neurorehabilitation, Qingdao University, Qingdao, Shandong, China; ^2^ Qingdao Medical College, Qingdao University, Qingdao, China; ^3^ Department of Interventional Radiology, The Affiliated Hospital of Qingdao University, Qingdao, Shandong, China

**Keywords:** NKT cell, γδ T cell, MAIT cell, meninges, brain injury, ischemic stroke, multiple sclerosis, neurodegeneration

## Abstract

The interaction between peripheral immune cells and the brain is an important component of the neuroimmune axis. Unconventional T cells, which include natural killer T (NKT) cells, mucosal-associated invariant T (MAIT) cells, γδ T cells, and other poorly defined subsets, are a special group of T lymphocytes that recognize a wide range of nonpolymorphic ligands and are the connection between adaptive and innate immunity. Recently, an increasing number of complex functions of these unconventional T cells in brain homeostasis and various brain disorders have been revealed. In this review, we describe the classification and effector function of unconventional T cells, review the evidence for the involvement of unconventional T cells in the regulation of brain homeostasis, summarize the roles and mechanisms of unconventional T cells in the regulation of brain injury and neurodegeneration, and discuss immunotherapeutic potential as well as future research goals. Insight of these processes can shed light on the regulation of T cell immunity on brain homeostasis and diseases and provide new clues for therapeutic approaches targeting brain injury and neurodegeneration.

## Introduction

Recently, the communication between peripheral immune cells and the brain has attracted the attention of an increasing number of researchers with the discovery of meningeal lymphatic vessels, a way for peripheral immune cells to infiltrate into the brain parenchyma and communicate with brain resident cells (1, 2). It is well known that the blood-brain barrier (BBB) is damaged upon brain injury, and peripheral immune cells enter the brain and release various cytokines or immune mediators to regulate the outcome of brain injury (3). Notably, under physiological conditions, some interfaces, including the meninges, choroid plexus and cerebrospinal fluid have been found to harbor various types of immune cells (4), which are involved in cognitive function (5), synaptic plasticity (6, 7) and neurogenesis (6). Thus, the immune system can communicate with the brain in both physiological and pathological conditions to modulate brain homeostasis and the progression of neurological diseases.

Unlike conventional T cells, which express an αβ TCR to recognize peptide antigens presented by major histocompatibility complex (MHC) molecules, unconventional T cells are a special group of T lymphocytes that mainly recognize a variety of self and non-self-antigens presented by nonclassical MHC molecules (8, 9). These unconventional T cells include natural killer T (NKT) cells, γδ T cells, mucosal-associated invariant T (MAIT) cells, and other rare subsets (10). Despite the limited number of unconventional T cells in organs of the body, defects or deficiencies of these T cells play important roles in various diseases, such as cancer, autoimmune diseases, and infectious diseases (11, 12). Notably, unconventional T cells were reported to be involved in both brain homeostasis (13, 14) and a variety of neurological diseases (15). Therefore, understanding the functions and molecular mechanisms of unconventional T cells in brain homeostasis and neurological diseases may provide clues for developing new therapies to maintain brain health.

In this review, we describe the classification and effector functions of unconventional T cells, including NKT cells, MAIT cells and γδ T cells; summarize the current reports on the functions of unconventional T cells in brain homeostasis, brain injuries caused by cerebral ischemic stroke, traumatic brain injury (TBI) and autoimmune-related multiple sclerosis, and neurodegenerative diseases; and discuss the immunotherapeutic potential of these cells as well as future goals of studies.

## Classification and function of unconventional T cells

**Figure 1:**
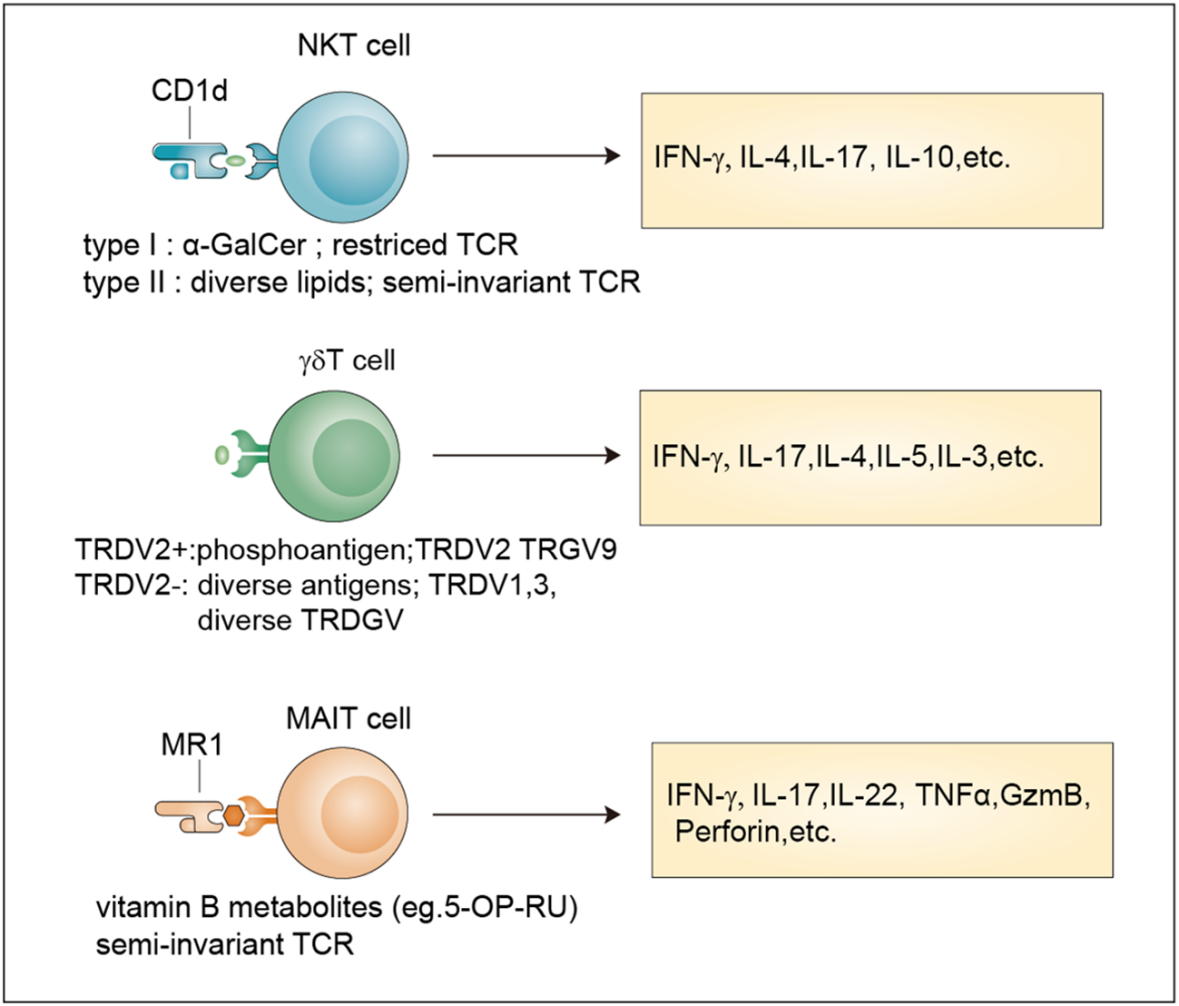
Classification and effector functions of unconventional T cells. Antigens, TCR properties and released-cytokines of NKT cells, γδ T cells and MAIT cells. NKT cells recognize antigens presented by Cd1d through an invariant αβ TCR. Type I NKT cells recognize the lipid antigen α-GalCer, while Type II NKT cells are reactive to more diverse lipid antigens. TRDV2^+^ cells and TRDV2^-^ γδ T cells have been defined based on their TCR δ-chain V region usage. TRDV2^+^ γδ T cells recognize phosphoantigens and TRDV2^-^ γδ T cells recognize more diverse antigens. MAIT cells are reactive to a limited array of microbe-derived vitamin B metabolites with a semi-invariant TCR. All these unconventional T cells release multiple cytokines after activation. α-GalCer, α-galactosylceramide.

Unlike conventional T cells which recognize peptide antigens presented by classical MHC molecules, unconventional T cells recognize diverse nonpeptide antigens through nonclassical MHC molecules. These unconventional T cells mainly include MAIT cells, NKT cells, and γδ T cells, which are generated from the thymus. Although far less numerous than conventional αβ T cells, unconventional T cells have unique effector and regulatory roles and are often described as a bridge between innate and adaptive immunity (**Figure 1**).

Compared with conventional T cells, unconventional T cells recognize a broader spectrum of antigens, including self-antigens and non-self antigens, such as peptides recognized by conventional T cells, conserved lipids and metabolites derived from bacteria. The recognition of these diverse antigens enables them to regulate multiple levels of cellular immune responses (16). In addition, unconventional T cells often localize in tissues, especially mucosal interfaces, which help maintain tissue homeostasis and repair (11, 17). Moreover, unconventional T cells, such as NKT cells and MAIT cells, acquire their effector functions in the thymus and can be quickly activated in a similar way to innate immune cells after encountering antigens (9). These properties endow unconventional T cells with diverse functions that expand and complement the function of traditional innate and adaptive immune cells in protective immunity, barrier function, and tissue healing (**Table 1**).

**Table 1:** Comparison between innate immune cells, conventional T cells and unconventional T cells.

	Innate immune cells	Conventional T cells	Unconventional T cells
Cells types	monocytes, macrophages, dendritic cells, neutrophils, basophils, eosinophils, NK cells, mast cells, and ILCs	αβ T cells	Mainly NKT cells, MAIT cells and γδ T cells
Recognition	Non-self structures (PAMPs), or danger or damage-associated molecules (DAMPs) are recognized by PRRs	Peptide antigens presented by MHC-I or MHC-II on APCs are recognized by TCRs	Self-antigens and microbial extended self and non-self-antigens are recognized by TCRs
Activation	PRRs activation dependent, very quick	TCR activation dependent, relatively slow	Innate-like activation modes, very quick
Function	primary line of defense against pathogens	adaptive protective immunity against pathogens	Tissue homeostasis, primary line of defense

ILCs, Innate lymphoid cells; PAMPs, pathogen associated molecular patterns; DAMPs, damage or danger associated molecular patterns; PRRs, pattern recognition receptors.

### Classification

NKT cells are generated from the CD4^+^CD8^+^ double-positive (DP) cells of the thymus, which represent a specialized subset of T cells that simultaneously express TCR and NK cell lineage markers. Two broad subsets, namely, type I NKT cells and type II NKT cells, have been defined (9). Typical type I NKT cells, also known as invariant NKT cells (iNKT cells), harbor an invariant TCRα chain (Vα14-Jα18 in mice and Vα24-Jα18 in human) coupled to a limited array of TCRβ chains that reactive to lipid antigens (α-GalCer) presented by CD1d (18). Currently, the endogenous ligands of iNKT cells remain unknown and need to be identified. In contrast, type II NKT cells have a more diverse repertoire than iNKT cells and can recognize a broader range of antigens, such as glycolipids, phospholipids, and hydrophobic antigens (19–21). The brain has abundant lipid content (22) indicating their involvement in NKT cell activation.

Similar to NKT cells, MAIT cells are also selected by DP cortical thymocytes and show limited TCR diversity that consists of an invariant α-chain (Vα 7.2-Jα 33 in humans; Vα 19-Jα 33 in mice) pairing with limited β chains (β2 or Vβ13 chain in humans; Vβ8 or Vβ6 chain in mice) (9). Accordingly, antigens recognized by MAIT cells are limited and are mainly vitamin B metabolites including 6-formylpterin, folic acid and several ribityllumazines and pyrimidine-based intermediates presented by MR1, a β2-microglobulin-associated antigen-presenting molecule (23).

Different from MAIT cells and NKT cells, γδ T cell subsets are generated from CD4^–^CD8^–^ double negative (DN) thymocytes and express a TCR γ-chain paring with a TCR δ-chain that exhibits limited TRGV and TRDV gene-segment usage (9). A unique feature of murine γδ T cells is that different tissues express distinct Vγ segments regarding the Vγ chains (24). In humans, two categories of γδ T cells including TRDV2^+^ cells and TRDV2^-^ cells have been broadly defined according to their TCR δ-chain V region usage, with the former coexpressing TRGV9 and the latter pairing with an array of TRGV genes (25). For many years, many researchers have tried to identify new γδ TCR ligands, and the involvement of butyrophilins or the recognition of MHC-like molecules has been revealed (26, 27). However, considering their important roles in both health and diseases (28, 29), large-scale screening methods based on current technologies such as the CRISPR/Cas9 system for further ligand identification are still needed.

### Effector functions

After TCR-mediated activation, unconventional T cells are activated to secrete cytokines, exert cytotoxic effector functions, and undergo proliferative expansion. Unlike conventional T cells, unconventional T cells can rapidly become effector cells and produce cytokines within minutes to hours upon antigen activation, which is an innate-like property (9). More importantly, unconventional T cells including NKT cells and MAIT cells acquire effector functions in the thymus during early development which requires the expression of transcription factor PLZF and costimulatory signals mediated by signaling lymphocyte activation molecules (SLAM) (9, 30). This feature confers unconventional T cells the ability to migrate to peripheral tissue early in life and rapidly respond to stimuli (11).

In response to antigen-mediated activation, NKT cells quickly secrete large amounts of proinflammatory cytokines, such as interleukin 17 (IL-17) and interferon-γ (IFN-γ), and anti-inflammatory cytokines, such as IL-4 and IL-10 (31). By comparing with the classification methods of conventional CD4^+^ T cells, researchers divide NKT cells into different subsets namely Th1-like NKT cell (NKT1), Th2-like NKT cell (NKT2), Th17-like NKT cell (NKT17) and Tfh-like NKT cell (NKTfh) based on the transcription factors and cytokines co-expressed by NKT cells (32, 33). Their differentiation potential is determined by the activation mechanism, which, in turn, determines the effects on the progression of diseases such as cancer, tissue injuries, autoimmune disease, and infection (34).

MAIT cells can be activated in two different ways: TCR-dependent and TCR-independent ways (35). TCR-dependent activation requires the presentation of microbe-derived intermediates by MR1 to TCR in cooperation with costimulators such as CD28 or some cytokines (36). After activation, MAIT cells secrete proinflammatory cytokines, mainly IL-17. TCR-independent activation is induced by IL-18 in combination with type I interferons, IL-12, or IL-15 and potentiated by TL1A, a membra of the TNF-superfamily. This form of activation stimulates a modest cytokine production dominated by IFN-γ instead of IL-17 (35, 37). They are involved in responses to infections, cancer, and tissue repair (35).

Upon activation by extracellular viruses, bacteria, fungi, or intracellular pathogens, γδ T cells can quickly produce various cytokines, such as tumor necrosis factor (TNF), IL-17, IFNγ, IL-4, IL-13 and IL-5 to protect the host against pathogens (38). In addition, they also exert cytotoxicity against transformed or infected cells by enhancing the expression of death-inducing receptors such as CD95 and TNF-related apoptosis-inducing ligand receptors (TRAILR) or releasing cytotoxic effector molecules, such as granzymes and perforin (39). Moreover, some immunosuppressive cytokines such as IL-10 and transforming growth factor-β (TGFβ), can be produced by γδ T cells to manipulate innate and adaptive immunity (38).

After brain injury and neurodegeneration, many damage-associated molecular patterns (DAMPs) are released and these unconventional T cells can be recruited into the brain and activated to secrete cytokines and growth factors that may regulate innate and adaptive immune cells or directly interact with neurons to regulate the outcome of the disease. However, some interesting questions still exist. For example, what types of brain-specific antigens do these unconventional T cells recognize? Are specific subsets of these unconventional T cells generated during brain homeostasis or after disease progression? How do these unconventional T cells interact with brain-resident cells?

## Unconventional T cells in brain homeostasis

Compared with other organs, the brain has a unique response to environmental stimuli owing to the existence of the BBB and the inability of neurons to self-renew (40, 41). Both intrinsic neuronal properties and external environmental factors determine the neuronal excitability and homeostasis of the brain (42). As an external factor, peripheral immune cells contribute to the homeostasis of many tissues (43), whereas their contribution to brain homeostasis and function has been largely ignored for years owing to the sporadic presence of peripheral immune cells in the brain parenchyma under homeostatic conditions.

**Figure 2:**
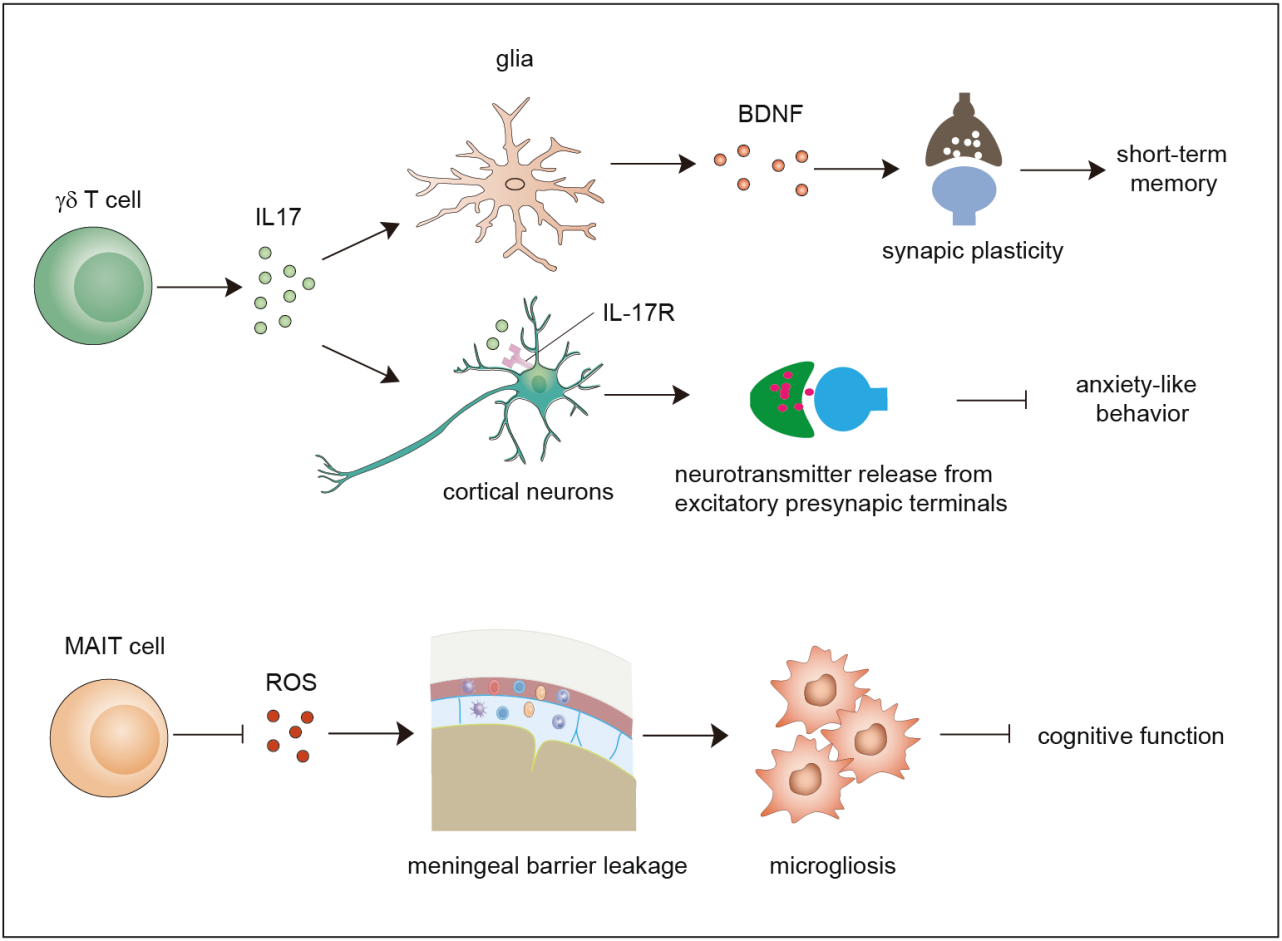
Functions of γδ T cells and MAIT cells in brain homeostasis. Meningeal γδ T cells mainly produce IL-17. The released IL-17 cytokine can on one hand act on glial cells to trigger BDNF expression to improve synaptic plasticity which potentiates short-term memory, and on the other hand directly act on neurons through IL-17R to enhance the release of neurotransmitters from excitatory presynaptic terminals to avoid anxiety-like behavior. MAIT cells express antioxidant molecules to preserve meningeal barrier homeostasis and integrity and protect the brain from cognitive decline. IL-17, interleukin 17; BDNF, brain-derived neurotrophic factor.

Unlike the relatively immune-privileged brain parenchyma, the surrounding meninges are populated by many resident immune cells and serve as an important interface with the peripheral immune system. Recent studies have updated our knowledge of meningeal immunity regarding how the complicated immune niche of the meninges affects brain functions and plasticity in a disease-free context (44) as well as in brain disorders. It is well known that conventional T cells play key roles in maintaining brain functions, including spatial learning (5, 45), social behaviors (46) and sensory responses (47). Given the importance of unconventional T cells, their regulation is recognized as a factor in the maintenance of brain homeostasis (**Figure 2**).

Early studies used polymerase chain reaction (PCR) analysis to show that diverse types of γδ TCRs are expressed in normal brain tissue (48). Currently, it is widely agreed that a large number of γδ T cells exist in the meninges instead of the normal brain parenchyma. Studies showed that γδ T cells derived from the fetus infiltrate the meninges after birth through the CXCR6–CXCL16 axis and are maintained by slow self-renewal (7, 49). Interestingly, these γδ T cells display sufficient IL-17-producing capability that is independent of inflammatory signals and are more activated than peripheral cells in the spleen. Ribeiro et al. proposed that T-cell receptor engagement in combination with signals derived from commensal microbes contributes to IL-17A production. Functional studies using IL-17-knockout mice and γδ TCR-knockout mice demonstrated that meningeal IL-17-producing γδ T cells are required for short-term memory maintenance and plasticity of glutamatergic synapses. IL-17 promotes the secretion of brain-derived neurotrophic factor (BDNF) from glial cells in the mouse hippocampus, which facilitates the synaptic plasticity of neurons to maintain short-term memory (7). Meanwhile, another study demonstrated that the release of IL-17 promotes anxiety-like behaviors by contributing to the proper release of neurotransmitters from the excitatory synapses of medial prefrontal cortex (mPFC) neurons in an IL-17R-dependent manner (49). These two studies found that IL-17-producing γδ T cells promote brain homeostasis, although some questions remain to be resolved. For example, how do cytokines of meningeal origin function in the parenchyma? How do meningeal γδ T cells sustain the production of IL-17? Does IL-17 act in an IL-17R-dependent manner?

Recently, a study showed that MAIT cells can be found in the meninges but are absent in brain parenchymal regions, and they exhibit high expression of antioxidant proteins that promote the maintenance of meningeal MAIT cells. MAIT cell deficiency results in reactive oxygen species (ROS) accumulation and barrier leakage, which results in inflammation of the brain parenchyma and cognitive decline in the brains of 7-month-old mice (50). Thus, MAIT cells contribute to the maintenance of physiological neural function by preserving the integrity of the meningeal barrier as well as meningeal homeostasis (14, 50, 51). These reports indicate that both γδ T cells and MAIT cells are involved in maintaining brain homeostasis. Whether NKT cells also exist in the meninges and contribute to brain homeostasis is a matter that needs to be investigated in the future.

## Unconventional T cells in brain injury

Both acute brain injuries, including cerebral ischemic stroke, hemorrhagic stroke, and brain traumatic injury, and chronic brain injuries resulting from autoimmune-related attacks, such as multiple sclerosis, entail a high risk of mortality and disability. The recognition of DAMPs released from damaged brains initiates strong inflammatory responses in the brain parenchyma, which promotes the recruitment of peripheral immune cells, such as dendritic cells, neutrophils, and T cells to the region of injury (3, 52). Many reports have demonstrated that immune responses-induced by peripheral immune cells regulate the pathogenesis of brain injury as well as long-term functional recovery (3, 53). Therefore, the functional properties of unconventional T cells in acute and chronic cerebral injury cannot be ignored.

### Ischemic stroke

Ischemic stroke is a devastating brain injury with high mortality and morbidity worldwide. Early reperfusion by intravenous alteplase and thrombectomy remains an effective treatment but leads to secondary brain damage owing to ischemia/reperfusion injury. Therefore, there is an urgent need to understand the mechanism and alleviate brain damage (54). Multiple studies have elucidated the roles of peripheral T cells in various stages of ischemic stroke (55–57), which may provide novel perspectives on immunotherapies for ischemic stroke. Previous studies have revealed the infiltration of unconventional T cells in the brain parenchyma (15, 58) and alternations in the number of unconventional T cells in the peripheral blood (59), indicating the involvement of unconventional T cells in ischemic cerebral injury (**Table 2**).

**Table 2:** Direct evidence of unconventional T cells in ischemic stroke.

Ischemic stroke models	Study methods	Cell type	Effects on disease progression	Reference
tMCAO	CD1d^-/-^ mice	type I and II NKT cell	Fail to influence infarct volume, facilitate post-stroke immune suppression in CD1d^-/-^ mice	60, 61
tMCAO	α-Galcer injection	type I NKT cell	α-Galcer injection inhibits post-stroke infection	60
pMCAO	α-Galcer or its analog injection	type I NKT cell	α-Galcer injection increases infarct volume and aggregate brain edema	62
tMCAO	MR1^-/-^ mice	MAIT cell	MAIT cell deficiency leads to a reduction of infarction and an improvement of neurological dysfunction.	63
tMCAO	DNT cell transfer to Rag1^-/-^ mice	DNT cell	DNTs enhance neuroinflammatory responses and exacerbate ischemic brain injury by modulating the FasL/PTPN2/TNF-α signaling axis.	64
tMCAO	TCRγδ KO mice	γδ T cell	γδ T cell ameliorated the I/R injury	65
tMCAO	IL-17A blocking antibody	IL-17-producing γδ T cell	IL-17 neutralization reduces neutrophil invasion and protects mice from ischemic injury	66
tMCAO	CCR6^-/-^ mice	IL-17- producing γδ T cell	CCR6 triggers the infiltration of IL-17-producing γδ T cells to exacerbate brain injury	67
tMCAO	TCRγδ KO mice	γδ T cell	γδ T cells-deficiency alleviates motor function injury and reduces the volume of brain infarction	68

tMCAO, transient middle cerebral artery occlusion; pMACO, permanent middle cerebral artery occlusion.

As a bridging system between innate and adaptive immunity, iNKT cells accumulate in the ischemic hemisphere or peripheral blood in mice or rats subjected to middle cerebral artery occlusion (MCAO) or patients suffering stroke. In addition, these infiltrating iNKT cells primarily produce Th2-like cytokines, including IL-10 and IL-5, and few Th1-like cytokines, such as IL-12 and IFN-γ (15, 69–71). The influence of iNKT cells on the severity of cerebral ischemic stroke depends on the study methods and mouse model used. Administration of the specific iNKT cell-activating ligand α-GalCer, compared with a vehicle control, increases infarct volume and improves neurological deficiency and brain edema after pMCAO (62). Nevertheless, depletion of iNKT cells by using Cd1d^-/-^ mice fails to alter infarct volume after tMCAO (60, 61) but the mice showed greater pulmonary damage, decreased survival rate, and more infiltration of neutrophils, a characteristic of poststroke pulmonary infections. Within the currently feasible conditions of clinical treatment, stroke-induced immunosuppression is detrimental to the human body, as it increases the risk of poststroke infections, especially infections of the lung and urinary tract (55, 56, 72). Several studies provide a mechanistic explanation of this phenomenon, and T lymphocytes are involved in modulating poststroke-induced immunosuppression as well as the subsequent infections (73). Interestingly, upon ischemia, iNKT cells exhibit restricted crawling and produce more Th2-like cytokines and fewer Th1-like cytokines, a set of conditions that are present in patients with acute ischemic stroke (60, 71). Selective immunomodulation of iNKT cells with α-GalCer or propranolol, a nonspecific β-adrenergic receptor blocker, promotes the production of proinflammatory cytokines and restricts pulmonary infection after ischemic stroke (60). Therefore, iNKT cells are involved in poststroke infections, and modulating iNKT cell function with the corresponding ligand may provide a potential way to alleviate stroke-associated infections. Thus far, it remains unknown how iNKT cells are activated after ischemia, considering their restricted recognition of lipid antigens.

γδ T cells also accumulate in the peripheral blood or brain parenchyma of stroke patients as well as MCAO-subjected mice as early sensors of ischemic brain injury (65–67, 74). Notably, brain-infiltrating γδ T cells are the main sources of IL-17, one of the main neuroinflammatory cytokines released after ischemia (65). The migration of IL-17-producing γδ T cells into the ischemic hemisphere relies on C-C chemokine receptor type 6 (CCR6) (67). Genetic depletion or antibody blockade of γδ T cells or genetic blockade of IL-17^+^ γδ T-cells infiltration leads to improved neurological outcomes by regulating neutrophil recruitment and BBB integrity (65–68). Interestingly, alterations in the intestinal flora-induced by antibiotic treatment substantially alleviated ischemic brain damage in mice. The priming of intestinal dendritic cells (DC) by altered commensal bacteria results in local Treg cell expansion and IL-17^+^ γδ T cells suppression, which further leads to decreased migration of intestinal IL-17-producing γδ T cells into the brain meninges and protects the brain against ischemic injury (75). This study confirms the vital regulation of the microbiota–gut–brain axis in response to cerebral ischemic stroke. Clinical and experimental studies indicate that gut microbial composition is altered after ischemia (76–79); stroke risks and stroke outcomes are influenced by the composition of the gut microbiota (75, 77, 80). For instance, antibiotic treatment or fecal matter transplantation from young mice into aged mice all improve stroke outcomes by changing gut microbiota (81). Although long-term regulation is mainly mediated by the vagus nerve and by metabolites, immune cells such as γδ T cells also contribute to the modulatory effect of microbiota on brain injury (75, 82).

Likewise, the infiltration of MAIT cells is increased early after ischemic stroke. MR1 deficiency or suppression of MAIT cell activation using a suppressive MR1 ligand decreases infarct volume, improves neurological deficits, and reduces microglial activation as well as the production of proinflammatory cytokines (63), which indicates that the use of MAIT cells may serve as a new method for the treatment of acute ischemic stroke by regulating neuroinflammation.

Apart from iNKT cells, MAIT cells and γδ T cells, DNT (CD3^+^CD4^-^CD8^-^) cells are another unconventional T-cell subset that shows positive expression of T lymphocyte antigen receptors but negative expression of CD4 and CD8 markers (83). One report showed that DNT cells are involved in poststroke neuroinflammation in patients and animal models (64). After stroke, DNT cells accumulate in peripheral blood as well as in the ischemic penumbra. The infiltrating DNTs in the brain parenchyma exacerbate brain injury and promote neuroinflammatory responses via the FasL/PTPN2/TNF-α signaling axis. Blockade of the above pathway restricts DNT-mediated neuroinflammation and improves the outcomes of stroke (64). Many studies have observed and reported functional subsets of NKT cells, γδ T cells or MAIT cells based on CD4 and CD8 expression, including DP or DN phenotypes; thus, CD4/CD8 DP or DN cells are likely to be a mixture of many cell subpopulations with this phenotype, in which some functional subsets of NKT, γδ T cells or MAIT cells might be included (84–89). Therefore, whether these DNT cells include NKT cells, γδ T cells or MAIT cells is a matter that still needs further investigation.

### Hemorrhagic stroke

Hemorrhagic stroke, which accounts for 10-20% of all strokes has considerable morbidity and mortality, and the most common type of hemorrhagic stroke is intracerebral hemorrhage (ICH) (90). Recent reports have shown that neuroinflammation and host immune responses contribute to the pathophysiology of hemorrhagic stroke (91, 92). As the BBB is also damaged during hemorrhagic stroke, the role of brain parenchyma-infiltrating peripheral immune cells has been studied (93), although their role is slightly more limited in hemorrhagic stroke than ischemic stroke. Previous studies demonstrated that both CD4^+^ T and CD8^+^ T lymphocyte numbers are increased in murine models of intracerebral hemorrhage, which are associated with inflammatory damage during hemorrhagic stroke (94, 95). Regulatory T (Treg) cell, an immunosuppressive T-cell subset, exerts neuroprotective effects in ICH and subarachnoid hemorrhage (SAH) (96, 97). At present, the known role of unconventional T cells in hemorrhagic stroke is very limited. However, considering the regulatory roles of unconventional T cells in ischemic stroke, the function of unconventional T cells in hemorrhagic stroke is also worth investigating in the future.

### Traumatic brain injury

Traumatic brain injury (TBI) is another class of brain injury that involves complex neurological processes including acute molecular alternations and chronic neurocognitive decline (98, 99). In peripheral blood from trauma patients, circulating NKT cell and MAIT cell numbers are reduced (100), and NKT cells show reduced proliferative capacity and IFNγ production in response to α-GalCer stimulation, which may affect disease outcomes (100). Mice with γδ T cell-deficiency exhibit alleviated inflammation in the acute phase of experimental TBI and show improved cognitive function in the chronic phase of TBI. In addition, different subsets of γδ T cells play distinct roles in TBI-induced brain injury. Vγ1 γδ T cells infiltrate the brain parenchyma and produce IFN-γ and IL-17 to trigger microglia-mediated neuroinflammation, whereas Vγ4 γδ T-cell subsets secrete TGF-β to maintain microglial homeostasis and reduce TBI-induced brain injury (101). The roles of different unconventional T-cell subsets in TBI or other forms of brain injury and their mechanism of action remain unknown.

### Autoimmune-induced chronic brain injury

Multiple sclerosis (MS) is a chronic autoimmune disease of the central nervous system (CNS) that causes irreversible neurological injuries by attacking the protective myelin sheaths of neurons (102, 103). Autoreactive T-cells mediated demyelination has been intensively investigated and is currently regarded as the main attack on the CNS in MS, but the initial cause of the disease needs investigation (104, 105). Understanding the mechanism of neuron-specific T-cell generation can provide an important target for effective treatment. Experimental autoimmune encephalomyelitis (EAE), a widely accepted animal model of MS used by researchers, shares some pathological features with clinical MS, including strong neuroinflammatory responses, massive demyelination and axonal loss (106). Therefore, studies based on EAE models can provide important clues for the clinical treatment of MS.

Several studies examined circulating NKT cell numbers and functions in MS patients. NKT cell numbers change differently in the blood of MS patients from different studies (107–110). In addition, distinct cytokine production profiles have been observed in NKT cells from MS patients. CD4^+^ NKT cells produce more IL-4 in RRMS (relapsing-remitting) patients than in patients with progressive MS or in healthy controls (111). However, NKT cells in secondary progressive MS patients display a proinflammatory response (112). Different types of MS or distinct stages of its progression may account for these conflicting findings.

**Figure 3:**
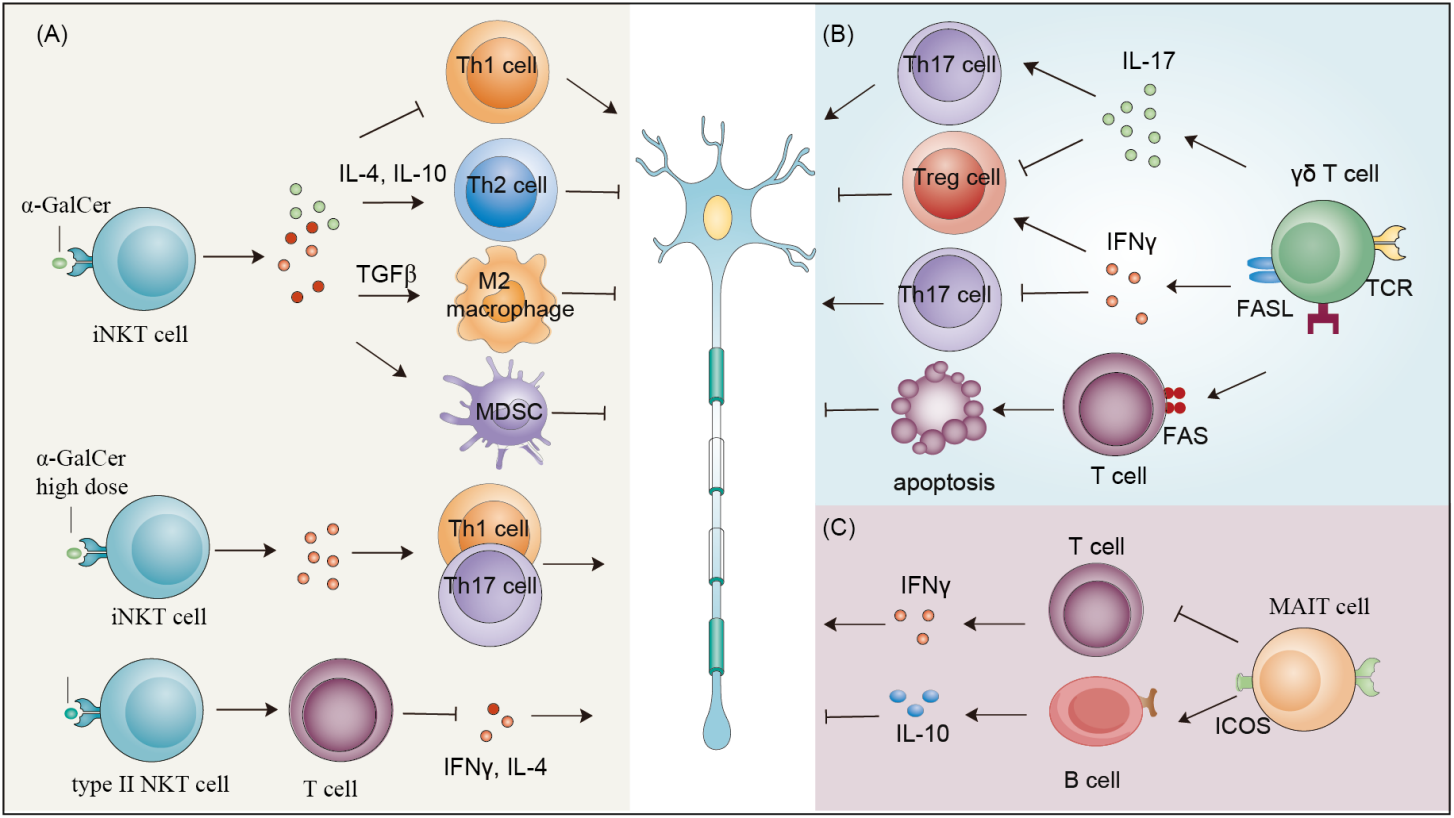
Effects of NKT cells, γδ T cells and MAIT cells on EAE. **(A)** Stimulating iNKT cells with α-GalCer or its analog can either reduce Th1 response or improve Th2 response, potentiate MDSC or M2 macrophage differentiation to improve EAE outcomes. High doses of α-GalCer selectively increase Th1 and Th17 responses to exacerbate EAE progression. Sulfatide activates type II NKT cells, which suppress IFN-γ and IL-4 production by pathogenetic antigen-reactive T cells and protect mice from EAE. **(B)** IL-17-producing γδ T cells exacerbate EAE progression by increasing inflammation by not only promoting Th17 cell function but also restraining the generation of Tregs. IFNγ-producing γδ T cells suppress the activity of Th17 cells and release chemokines to recruit Treg cells to reduce inflammatory signals. Moreover, γδ T cells can induce the apoptosis of encephalitogenic T cells through Fas/Fas ligand signaling and facilitate recovery from EAE. **(C)** MAIT cells alleviate the progression of EAE by decreasing inflammatory mediators and increasing IL-10 production. MDSC, myeloid-derived suppressor cell; EAE, experimental autoimmune encephalomyelitis; Treg, regulatory T cells.

The implication of NKT cells in EAE is elusive, although it has been investigated by many studies (**Figure 3**). Elimination of NKT cells by using mice from distinct backgrounds or EAE models exhibits conflicting effects. Some studies have observed no obvious effects (113, 114), whereas some studies have observed disease exacerbation in CD1d-knockout mice (115) as well as in Jα18-deficient mice (114, 116, 117). Similarly, administration of α-GalCer or its analog to activate NKT cells also exhibits contrasting effects on the outcome of EAE. Most studies have shown that iNKT cells activation alleviate the Th1 response, improve the Th2 response (113, 114, 118) or potentiate MDSC (119) and M2 macrophage differentiation (116) to improve EAE outcomes (113, 114, 116, 118, 119). Surprisingly, high doses of α-GalCer selectively elevate Th1 and Th17 responses by interacting with CD1d expressed on T cells but not type I NKT cells to exacerbate EAE progression (120). Furthermore, immunization of α-GalCer in combination with myelin antigens potentiates EAE in B10.PL mice by promoting the Th1 response and preventing EAE in C57BL/6 mice by increasing IL-4 production (121). Therefore, the outcome of EAE relies on the downstream targets that NKT cells activate. In addition to α-GalCer, sulfatide, a major component of the myelin sheath, can activate type II NKT cells, which suppress IFN-γ and IL-4 production by pathogenetic antigen-reactive T cells and protect mice against EAE in a CD1d-dependent manner (122). Notably, the first-in-human study using the iNKT cell stimulatory drug (2S,3S,4R)-1-O-(α-D-Galactopyranosyl)-N-tetracosanoyl-2-amino-1,3,4-nonanetriol (OCH) based on previous mouse studies (118, 123) demonstrated the immunomodulatory effects of iNKT cells on MS patients, providing valuable information for the clinical application of OCH as a potential therapy for MS (124). Moreover, some currently available drugs targeting MS alter the properties of NKT cells (15).

γδ T cells accumulate to a significant extent in the brains of MS patients (125), and their frequency is also increased in the peripheral blood and cerebrospinal fluid of MS patients (126–128). Further phenotypical and functional analysis showed that circulating γδ T cells in relapsing-remitting multiple sclerosis (RRMS) patients have a reduction in the circulating CCR5^+^ γδ T-cell subset with decreased EOMES and granzyme B mRNA abundance and increased production of IFN-γ (129). To better characterize the infiltrated γδ T cells, TCR repertoire analysis was performed, and oligoclonal expansion of γδ T cells was observed, suggesting their responses to common antigens (127, 130, 131). Accordingly, common antigens recognized by γδ T cells were reported. A subpopulation of γδ T cells in MS lesions respond to heat-shock proteins, including HSP70 (132) and HSP65 (133). In addition, sulfatide, a myelin-derived antigen, can also be recognized by γδ T cells in the MS brain indicating that lipid antigens can be presented to γδ T cells after MS (134). Thus, the number, specificity, and function of γδ T cells altered in MS patients, although the variation may be distinct in different types and progression stages of MS.

The specific subsets and effector function of γδ T cells in EAE have been investigated extensively (**Figure 3**). Several studies have suggested that diverse γδ T subsets are present in the brain in EAE. During the early stage of EAE, brain parenchyma-infiltrating γδ T cells harbor a restricted TCR repertoire, including Vδ1, Vδ5, Vδ4, Vγ1–3, and Vγ6, while during later phases, Vγ and Vδ are widely distributed in the brain (135, 136). During disease progression, peripheral γδ T cells can be induced to produce cytokines, especially IL-17, in response to stimulation with IL-23 and IL-1β, which are released by dendritic cells and microglia in EAE (137, 138). IL-23 or IL-15-activated γδ T cells exacerbate EAE progression by increasing inflammation by not only promoting Th17 cell function but also restraining the generation of Tregs (139–141). However, IFN-γ-producing γδ T cells suppress the activity of Th17 cells and release chemokines to recruit Treg cells to reduce inflammatory signals (139). Moreover, γδ T cells can induce the apoptosis of encephalitogenic T cells through the Fas/FasL signaling pathway and potentiate functional recovery after EAE (142). Notably, encouraging data have been obtained in several clinical trials targeting IL-17 in relapsing-remitting MS patients (143, 144). As an important cytokine released from T cells, IL-17 can, on one hand, directly inhibit oligodendrocyte progenitor cell (OPC) differentiation and exert toxic effects on OPCs and oligodendrocytes (145–147), altering myelin stability and regeneration after MS, and, on the other hand, regulate immune cell activation which indirectly affects myelination. Therefore, in the early stage of EAE, γδ T cells are more pathogenic than protective given that γδ T cells abundantly and predominantly release IL-17 in the brain.

As with NKT cells and γδ T cells, many studies have confirmed the infiltration of MAIT cells especially CD8^+^ MAIT cells, into the brain upon activation by IL-18 or IL-23 triggered by enriched fungal content in the gut as observed through immunofluorescence staining or TCR repertoire analysis (148–152). Their number and function in the peripheral blood remain controversial, as one study observed no obvious difference between relapsing-remitting MS patients and healthy volunteers (153), while others showed a reduced frequency in relapsing-remitting MS patients (149, 154, 155). Deficiency of MAIT cells in mice exacerbates EAE progression by increasing inflammatory mediators and reducing the production of anti-inflammatory cytokine IL-10 (156) (**Figure 3**). Future studies are needed to investigate the roles of MAIT cells during different stages of multiple sclerosis.

In addition to MS, another autoimmune condition of interest is autoimmune encephalitis, a group of diseases with subacute or progressive inflammation of the brain that results in various neurological or psychiatric symptoms owing to immune cell-produced antibodies against neuronal cell surface or synaptic antigens (157, 158). Previous studies have implicated that autoimmune encephalitis usually occurs when viruses or tumors cause neuronal proteins to be exposed to the immune system. Conventional T-cells and B-cells-mediated immune responses are responsible for leading to this disease, although the released antigens or the first encountered immune cells determine which immune cells dominate in a certain type of autoimmune encephalitis (159–162). Currently, the role of unconventional T cells in autoimmune encephalitis remains unknown. Considering their recognition of diverse antigens and their rapid response after activation, their roles in autoimmune encephalitis are worth investigating.

## Unconventional T cells in neurodegeneration

Neurodegenerative diseases (NDDs) which include Alzheimer’s disease (AD), Parkinson’s disease (PD), Huntington’s disease (HD) and amyotrophic lateral sclerosis (ALS) represent a major threat to human health. Identifying the initial causes and molecular mechanisms underlying each disease is required for the effective treatment of neurodegeneration (163, 164). Emerging evidence has shown that peripheral immune cells infiltrate the brain and impact neurodegeneration (165). T cells are present in brain tissues of patients with neurodegeneration, although they are less abundant than the lymphocytes that infiltrate the brain in multiple sclerosis (166–168). Currently, it is believed that peripheral immune cells can impact the progression of NDDs, either after infiltrating into the brain or while staying in the periphery (165), and the roles of unconventional T cells in NDDs are under investigation (**Table 3**).

**Table 3:** Evidence of unconventional T cells in neurodegeneration.

Neurological Diseases (Model or Sample)		Study tools	Cell subsets	Effects on NDDs	Reference
**AD**	5XFAD mice	MR1-deficient mice (IF)	MAIT cell	MR1-deficient 5XFAD mice exhibit a reduced plaque burden than WT mice.	169
	3xTg-AD mice	IL-17 blockage (flow cytometry)	γδ T cell	IL-17-producing γδ T cells were observed to increase in the brain and the meninges of female, but not male mice, by using the 3xTg-AD mice model; Blocking IL-17 prevents short-term cognitive deficits at early stages of the disease by improving synaptic dysfunction.	170
**ALS**	ALS patients’ blood	flow cytometry	NKT cell	NKT cell (CD3^+^CD16^+^CD56^+^) number was significantly increased.	171
	mSOD1 mice model	α-Galcer injection (flow cytometry)	type I NKT cell	NKT cell number (NK1.1 Cd1d-tetramer) increases dramatically in ALS mice; Immunomodulation of NKT cells using α-GalCer prolongs the life span of SOD1 mice.	172
**PD**	PD patient’s blood	flow cytometry	γδ T cell	γδ T cell number was increased in the blood and CSF of the blood of PD patients compared with patients with other neurological diseases.	173
	PD patient’s blood	flow cytometry	γδ T cell	γδ T cell number was reduced in patients with Parkinson’s disease compared with age-matched health controls.	174, 175
	PD patient’s blood	flow cytometry	iNKT cell	The frequency and absolute number of iNKT cells are reduced in the blood of PD patients compared with healthy controls.	170

AD, Alzheimer’s Disease; PD, Parkins’s Disease; NDDs, Neurodegenerative diseases.

The number of γδ T cells in the peripheral blood varies, with one study showing an increased number (176) and one study showing a reduced number (174). The variation may result from the different controls the authors chose as the former study used the blood from patients with neurological diseases other than PD, while the latter used healthy subjects. CD4^+^ helper type 17 (Th17) and γδ (γδ17) T cells are two major classes of IL-17-producing cells, and have emerged as key players in the progression of diseases (75, 140). Elevated production of IL-17 in patients with PD and AD was observed (173, 177). Accordingly, Th17 cells have been revealed to exacerbate neuroinflammation and neurodegeneration in both PD and AD models (175, 178). Concomitant with the onset of cognitive decline, Brigas et al. observed that the meninges and brains of female mice but not male mice exhibit an accumulation of IL-17-producing γδ T cells by using the 3xTg-AD mouse model. More importantly, in the early stages of the disease, neutralization of IL-17 markedly prevents synaptic dysfunction independent of tau or Aβ pathology or BBB disruption to maintain short-term cognitive function (170). Several reports have shown that γδ T cells can be stimulated by heat shock proteins (HSPs) and when cells are subjected to environmental stress, they express increased amounts of intracellular HSPs (179, 180) indicating antigenic involvement in NDDs. ELISAs of the CSF of PD patients revealed increased levels of HSP65 and HSP70 compared with corresponding controls, while no difference was observed in the serum (181). Whether HSPs can act as antigens for γδ T cells in other NDDs needs further investigation. As the ligands of γδ T cells are more diverse than other unconventional T cells, it will be interesting to test whether the aggregated proteins in NDD can directly activate or repress γδ T- cell activation.

Considering the implication of neuroinflammation in neurodegeneration, the evidence of NKT cells in neurodegenerative disease is limited (182). Zhou et al. showed that the frequency and number of iNKT cells are decreased in the peripheral blood of PD patients compared with healthy control subjects (174). In addition, one study showed obvious accumulation of NKT cells in the peripheral blood of ALS patients (171). Moreover, the number of iNKT cells was observed to increase in the liver, spleen, and spinal cord of transgenic mouse models with ALS (172). Furthermore, α-GalCer-induced NKT cell activation was found to suppress astrogliosis and alleviate motor neuronal death in the spinal cord. Modulation of NKT cells initiates a cytokine shift in the liver and facilitates T cell recruitment into the spinal cord which extends the lifespan of mSOD1 mice (172). For NKT cells, it is technically difficult to directly test their existence *in situ* owing to the limited methods available to specifically label this subset. In addition, it remains unknown whether NKT cell deficiency affects NDD progression. Considering the high content of glycolipids in the brain tissue (183), more research is needed to gain better insight into NKT cells in other NDDs.

Similar to NKT cells and γδ T cells, MAIT cells play a limited role in NDDs is also limited. One study revealed an increase in MAIT cell numbers and elevation of activation in the brains of 5XFAD mice. More importantly, a significantly reduced plaque burden was observed in MR1-deficient 5XFAD mice compared with WT mice indicating the contributing role of MAIT cells in the development of AD pathology (169), although the detailed molecular mechanism is unknown.

### Potential clinical applications of unconventional T cells

The above-summarized roles of unconventional T cells indicate that modulating unconventional T cells in the following ways may have some clinical applications. First, the alternations of unconventional T cell subsets may be used as a diagnostic strategy. The frequency and number of NKT cells, MAIT cells and γδ T cells in the peripheral blood of patients suffering from brain injury (71, 107, 108, 153) and neurodegeneration (174, 176) significantly change. Monitoring the alternations in these cells during different stages of disease progression may provide an auxiliary way to diagnose disease progression. In addition, some drugs can be developed to block the function of these unconventional T cells. For example, abrogation of MAIT cell response can be achieved by administering MR1 blocking antibodies (184), while antibodies blocking BTN2 or BTN3 can be used to inhibit Vγ9Vδ2 T cell responses (185). Moreover, targeted immunotherapies are appreciated to manipulate unconventional T cells to modulate disease progression. On one hand, agonists of NKT cells, MAIT cells and γδ T cells, such as α-GalCer, 5-(2-oxopropylideneamino)-6-d-ribitylaminouracil (5-OP-RU) and aminobisphosphonates can activate the immune response under immune-suppressive conditions (25, 186, 187), and on the other hand, adoptive transfer of neuroprotective subsets of unconventional cells is another promising therapeutic strategy. With the progress that has been made in our understanding of the roles and mechanisms of unconventional T cells in brain injury and neurodegeneration, future clinical applications are within reach.

## Conclusion and perspectives

T lymphocytes play critical roles in both brain homeostasis and neurological diseases. Unconventional T cells, a special group of T lymphocytes, are beginning to have their roles in the brain characterized. In this review, we present evidence of the crosstalk between unconventional T cells and the brain in the maintenance of brain homeostasis, brain injury and neurodegeneration. It is conceivable that unconventional T cells in the meninges facilitate homeostatic regulation of the brain. Nevertheless, owing to different investigation methods and tools, conflicting evidence about the roles of these unconventional T-cell subsets in brain injury has been reported. Therefore, by better understanding the regulation and detailed mechanisms of detrimental effects versus beneficial effects in regulating brain injury and neurodegeneration, we believe effective and novel therapeutic targets will be discovered in the future.

Questions to be resolved in the future:

(1) How do meningeal unconventional T cells communicate with glial cells and neurons in the brain parenchyma under physiological and pathological conditions?
(2) Aside from MAIT and γδ T cells, do other unconventional T cells exist in meninges and regulate brain homeostasis?
(3) After injury, does the brain harbor some antigens that activate specific unconventional T-cell subsets?
(4) Do unconventional T cells directly interact with glial cells and neurons after infiltration during brain injury and neurodegeneration?
(5) How can single-cell sequencing techniques be used to define unconventional T-cell subset specificity in the brain under either physiological or pathological conditions?
(6) How can *in-situ* detection methods for unconventional T cells in the brain be improved?
(7) How can the ratio and function of these unconventional T cells be calibrated to maintain brain homeostasis and modulate disease progression?

## Author contributions

ML: Data curation, Investigation, Methodology, Software, Writing – original draft. ZZ: Data curation, Funding acquisition, Investigation, Methodology, Writing – original draft. YC: Conceptualization, Funding acquisition, Project administration, Resources, Supervision, Writing – review & editing.
